# Resolving the soluble-to-toxic transformation of amyloidogenic proteins: A method to assess intervention by small-molecules

**DOI:** 10.21203/rs.3.rs-2631727/v1

**Published:** 2023-03-06

**Authors:** Jyoti Ahlawat, Daisy L. Wilson, Ana Carreon, Mahesh Narayan

**Affiliations:** the University of Texas at El Paso (UTEP); the University of Texas at El Paso (UTEP); the University of Texas at El Paso (UTEP); the University of Texas at El Paso (UTEP)

**Keywords:** amyloid proteins, soluble-to-toxic conversion, gel electrophoresis

## Abstract

The soluble-to-toxic transformation of intrinsically disordered amyloidogenic proteins such as amyloid beta (Aβ), α-synuclein, mutant Huntingtin Protein (mHTT) and islet amyloid polypeptide (IAPP) among others is associated with disorders such as Alzheimer’s disease (AD), Parkinson’s disease (PD), Huntington’s disease (HD) and Type 2 Diabetes (T2D), respectively. Conversely, the dissolution of mature fibrils and toxic amyloidogenic intermediates including oligomers remains the holy grail in the treatment of neurodegenerative disorders. Yet, methods to effectively, and quantitatively, report on the interconversion between amyloid monomers, oligomers and mature fibrils fall short. For the first time, we describe the use of gel electrophoresis to address the transformation between soluble monomeric amyloid proteins and mature amyloid fibrils. The technique permits rapid, inexpensive and quantitative assessment of the fraction of amyloid monomers that form intermediates and mature fibrils. In addition, the method facilitates the screening of small molecules that disintegrate oligomers and fibrils into monomers or retain amyloid proteins in their monomeric forms. Importantly, our methodological advance diminishes major existing barriers associated with existing (alternative) techniques to evaluate fibril formation and intervention.

## Introduction

A hallmark feature of neurodegenerative disorders such as AD, PD, HD and T2D is the soluble-to-toxic conversion of disease-associated prion-like amyloidogenic proteins such as Aβ, α-synuclein, mHTT, and IAPP, respectively (1–7). The formation of mature fibrils from their soluble, monomeric counterparts is often the “end-point” of the amyloid-forming (amyloidogenic) trajectory. Fibril formation is essentially irreversible. Mature fibrils, which are rich in β-sheet content, are insoluble and therefore not easily amenable to structural studies.

Amyloid monomers are converted to mature fibrils via a sequential process that first results in the formation of dimers and/or neurotoxic oligomers (**Scheme 1**; 7).

Oligomers form proto-fibrils prior to the formation mature fibrils, which is a terminal process as aforementioned. A comparison of the kinetics of monomer consumption relative the fibril formation is important. A difference in the rate of monomer consumption relative to fibril formation suggests the presence of intermediates. A lag in the time to form mature fibrils is indicative of kinetically-trapped conformations (7). Quantifying the loss of monomers is essential for a detailed biophysical understanding of the amyloidogenic trajectory. After all, it is the most experimentally tractable of all species along the amyloid-fibril-forming pathway. The rate of monomer consumption informs us whether the ambient conditions are biased towards retaining the monomeric conformation or towards fibril formation. Measurement of the rate of monomer loss can be used to fine-tune ambient (fibril-forming) conditions either to intervene in the fibrillation or to promote it (say, for biophysical studies) (8). Comparison of the rate of monomer consumption with that of mature fibril formation facilitates the generation of a kinetic and quasi-structural roadmap of the process(es) by which soluble amyloids form insoluble aggregates.

Conversion of mature fibrils to their soluble monomeric counterparts is also indispensable for qualitative and quantitative evaluation of the efficacy by which small molecules may intervene (therapeutically or prophylactically) in amyloid-forming trajectories. Molecules such as tanshinone, brazilin and other aromatics along with specific carbon nano materials known as carbon quantum dots and graphene quantum dots have been instrumental in passivating amyloid monomers, remodeling oligomers, and dissolving mature fibrils (9–14). W.r.t. small molecule intervention, the ability to revert all non-monomeric intermediates including mature fibrils, to their soluble monomeric counterpart is key. Also critical is the ability to localize where along the fibril-forming trajectory that a small molecule intervenes is important for further advancing the candidacy of the said molecule (7).

Existing techniques to identify fibrils include dynamic light-scattering (DLS), fluorescence spectroscopy, advanced microscopy (AFM, TEM, HR-TEM, etc.), x-ray fiber diffraction, solid-state NMR, and EPR among others (15–20). While each technique offers specific advantages towards the detection of fibrils, they also require equipment that is not easily accessible, is expensive, and/or requires extensive sample preparation. Furthermore, the quantified conversion of mature fibrils to monomers by small-molecules is not easily realized using the aforementioned techniques.

Here, we demonstrate the use of gel electrophoresis to determine whether select small molecules revert mature fibrils to their soluble monomeric counterparts.

The advantages of our method over existing techniques is discussed.

## Method

### Gel Electrophoresis 12%

Gels were prepared as described elsewhere (21, 22). Briefly, for the running buffer, 1650 uL of water, 2000 uL of 30% acrylamide, 1250 uL of 1.5 M Tris (pH 8.8), 50 uL 10% ammonium persulfate and 2ul TEMED was combined in a 15 mL falcon tube and transferred to the slides. Later, the layering was completed using tertiary butanol. The gels were allowed to polymerize for about 20 minutes. The stacking solution containing 1550 uL of water, 250 uL of 30% acrylamide, 190 uL of 1.5 M Tris (pH 6.8), 15 uL ammonium persulfate and 1.5 ul TEMED in a 15 mL falcon tube was introduced into the gel on top of the running gel. The stacking gel was left to polymerize for 15 minutes and then stored at −4 °C until further use (using wet Kim-wipes covered with the aluminum foil).

#### Preparation of Lysozyme solutions

2 mg/mL of Hen-Egg White Lysozyme (HEWL; Sigma) solution in freshly made potassium phosphate buffer (20 mM, pH = 6.3, 3M Guanidinium Hydrochloride) was prepared in a 5 mL glass vial and kept in an incubator-shaker at 550 rpm for 6 hours at 58 °C. After 6 hours, (the contents of the glass vial were turbid), mature fibrils were visualized using Transmission Electron Microscopy (7).

### Loading of amyloid samples onto the gel

The aforementioned solution was dialyzed and added into 1.5 mL Eppendorf tubes and centrifuged (12,400 rpm for 15 minutes). The supernatant was collected in 1.5 mL Eppendorf tubes and DI water was added to the pellet and mixed well. 30 uL of the solution (including supernatant and pellet) was then transferred in separate 0.5 mL pre-labelled Eppendorf tubes. Later, 10 uL of 4X loading dye was added to 30 uL of supernatant and pellet solution. Monomeric solution of Lysozyme (2mg/mL) was prepared as a control and 30 uL was mixed with 10 uL of 4X loading dye. The samples were heated at 95 °C for 5 minutes and 20 uL of this solution was then loaded into the wells of the gel. The gel was then run for 85 minutes at 120V and 400 A. For staining-destaining, gels were removed from the glass slides and rinsed with water. Later, the gels were submerged in Coomasie staining solution overnight. The next day, destaining was performed s using 1:1 :0.2 ratio of water:methanol: acetic acid. Destaining was repeated thrice for 20 minutes each. After the third destaining wash, the gel was submerged in water to and an image was subsequently obtained using the Invitrogen iBright Imaging system.

#### Imaging of HEWL fibrils

For Transmission electron microscopy analysis, samples were suspended in deionized water and sonicated for 5–10 minutes before adsorption to carbon-coated Cu grids (Electron Microscopy Sciences, Hatboro, PA) followed by negative staining with 2.5% uranyl acetate. Excess stain was adsorbed with Whatman #1 filter paper and grids were air dried and viewed in a model H-7650 transmission electron microscope operated at 80 kV (Hitachi High-Technologies, Dallas, TX). Digital images were collected with an AMT XR 60 CCD camera system (Advanced Microscopy Techniques, Woburn, MA).

#### Fluorescence assays

Lysozyme samples were aliquoted for analysis after 0, 1, 2, 3, and 4 hours of incubation. Thioflavin T fluorescence (20 μM) was used to determine the fibril content of each sample in a DM45 Olis Spectrofluorometer using 450 nm and 480 nm as excitation and emission wavelengths, respectively.

#### Data Analysis

The obtained images of the gel using the iBright imaging system were analyzed using the Image J software. The data obtained from Image J were transferred to Origin Pro software and mean and standard deviation values are calculated for each band. The bar graph is plotted against Integrated Density vs Sample name.

## Results

**Figure 1:**
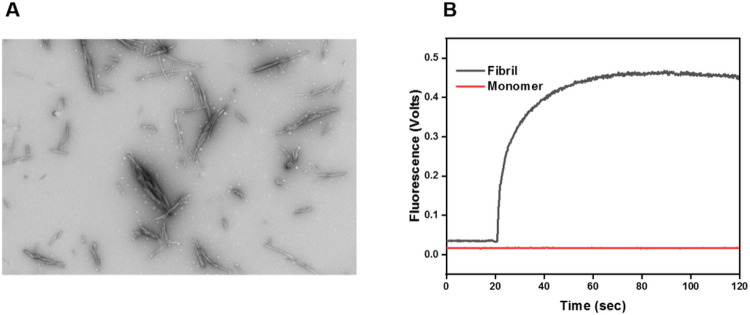
**A) TEM image of mature HEWL fibrils**. Both fibrils and smaller potentially proto-fibrillar structures are evident. **B) ThT assay for detecting amyloid fibrils**.Red: ThT added to a solution of lysozyme monomers. Black: ThT added to a solution containing mature HEWL fibrils.

Figure 1A is a representative TEM image of mature HEWL fibrils. The fibrils are needle-form and well-delineated in nature. The mature fibrils appear to be interspersed with smaller, potentially, proto-fibrillary aggregates. The data are in good agreement with previous literature (23). **1B** shows the increase in fluorescence emission that results when ThT is added (@ 20s) to a solution containing mature fibrils (black curve). The sharp and rapid increase in fluorescence intensity upon introduction of the fluorophore is indicative of ThT binding to fibrils (7, 23). The plateauing of the curve suggests that all fibril is either ThT bound or that there is no free ThT in solution even though there may be unbound fibrils. By contrast, the introduction of ThT to monomeric lysozyme (red curve) did not elicit any increase in fluorescence as anticipated.

**Figure 2:**
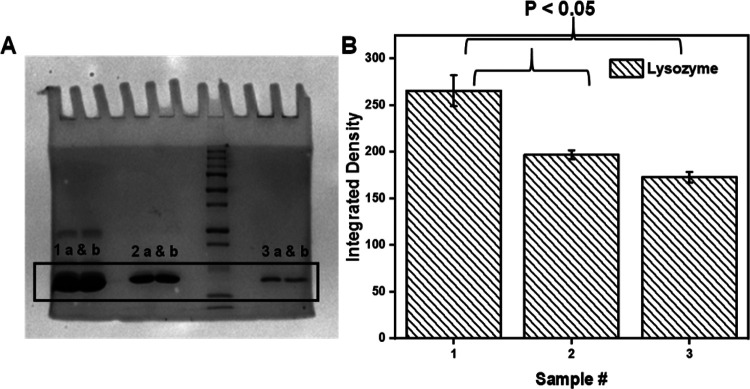
PAGE of HEWL solutions. **A)** Protein bands corresponding to HEWL monomers is detected across differing samples. 1a-b are monomers. 2a-b is supernatant (monomers remaining after HEWL fibrillation) and 3a-b is supernatant from the HEWL fibril pellet. **B)** The data are plotted for N = 2 where p < 0.05 was observed

We determined whether gel electrophoresis could be used to qualitatively discriminate between HEWL mature amyloid fibrils and its monomeric counterpart. Figure 2A is an image of a PAGE experiment where HEWL monomers (2: 1 a and b), the supernatant (**2A**: 2 a and b) from a centrifuged solution containing mature HEWL fibrils and a resuspended HEWL fibril pellet (**2A**: 3 a and b) were loaded onto the gel. The location of the bands correspond to the molecular weight of monomeric HEWL. Inspection of the band intensities reveals that compared to the sample exclusively containing HEWL monomers (**2A**: 1 a and b), there is a decrease in the intensity of HEWL monomers in sample (**2A**: 2 a and b) and a further attrition in its concentration when sampled from the fibril pellet (**2A**: 3 a and b). The mature fibrils do not enter the gel due to size-exclusion.

Figure 2B shows quantified results from the aforementioned experiment. Statistical significance was found between samples **2B**:1 and **2B**:2 and samples **2B**:1 and **2B**:3 indicating that PAGE can be used to quantify the soluble-to-fibril transformation of amyloid-fibril-forming proteins.

**Figure 3:**
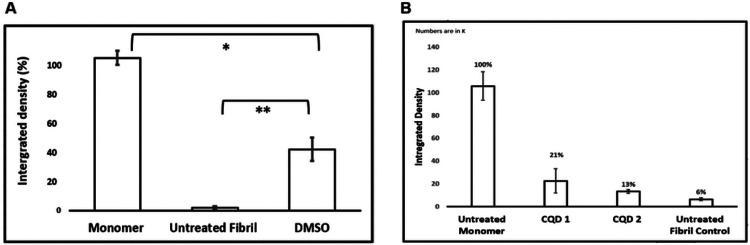
**A) HEWL fibrils treated with DMSO**. Quantification of band intensity corresponding to monomeric HEWL. Data shows the control sample (monomeric HEWL), untreated HEWL fibrils and DMSO treated HEWL fibrils. The data are plotted for N = 2 where p < 0.05 was observed. **B) HEWL fibrils treated with CQDs**. Quantification of band intensity corresponding to monomeric HEWL. Data shows the control sample (monomeric HEWL), HEWL fibrils treated with CQDs (1: ciric acid derived and 2: gelatin-derived) and untreated HEWL fibrils. The data are plotted for N = 2 where p < 0.05 was observed.

We tested whether small molecules and carbon nano materials revert HEWL fibrils to their soluble monomers. Dimethyl sulfoxide (DMSO) is known to dissociate amyloid fibrils (24, 25). Figure 3A shows an increase in the concentration of HEWL monomer, relative to untreated fibrils, when mature HEWL fibrils are exposed to DMSO. Furthermore, the difference in monomeric HEWL concentrations between DMSO-treated fibrils and untreated fibrils is statistically significant. The data indicate that the DMSO-driven reconversion of mature fibrils to their monomeric counterpart can easily be detected and quantified use gel electrophoresis. The (statistically significant) difference in monomeric HEWL concentration between the monomer control and the DMSO-treated fibrils is also notable. The fraction of monomer released from DMSO-treated HEWL fibrils reflects the small-molecule-driven fibril-to-soluble reconversion (at the small-molecule concentration). In principle, a small-molecule dose-response curve can be constructed to screen and rank candidate molecules.

Figure 3B shows results from HEWL fibril exposure to carbon quantum dots (CQD1: citric; CQD2:gelatinized carbon). Although there appears to be a CQD-dependent increase in soluble monomers relative to the untreated fibrils, the results were not statistically significant at the CQD dose used.

## Discussion

The soluble-to-toxic conversion of amyloid proteins such as Aβ, α-synuclein, mHTT among others is a critical milestone in the onset and pathogenesis of amyloid-specific neurodegeneraive disorders. Efforts to develop an understanding of this biophysical transformation are driven by spectroscopic and immunohistochemical tools. Nevertheless, access to instruments such as solid-state NMR, microscopes (TEM, HR-TEM, SEM, AFM), ATR-IR, DLS instruments and biochemical kits precludes routine studies of the process for many laboratories and investigators.

Even if high-resolution microscopes are accessible, extensive sample preparation protocols, analyses times and availability of very specific technical/instrumentation expertise are barriers that still need to be overcome. Finally, and critically, higher-resolution structural techniques are not amenable to quantification and kinetics measurements. As previously noted, quantification of oligomers and fibrils formed from soluble monomers and, perhaphs more importantly, the reverse process is important for advancing biomedical intervention. The *in vitro* screening of small-molecules that intervene in amyloidogenesis precedes testing in preclinical models.

Optical methods such as DLS or fluorescence using ThT or Congo red to identify fibrils are frequently confounded by interference from small-molecule fluorescence (26). Others techniques such as solid-state NMR, are not amenable to easy use, lack access, and fail to satisfactorily quantify the interconversion between the monomeric amyloid, its intermediates and the mature fibril. Often, necessary sample preparation conditions do not recapitulate solution conditions.

Through several inroads, the method described here reduce barriers towards the study of amyloidogenesis which has traditionally involved elaborate sample preparation, mounting of “dried” samples, expensive instrumentation and protracted sample analyses times (16, 17). Even though the technique is chemically and structurally “low-resolution” in nature, it provides a rapid, facile and inexpensive mechanism by which to quantify the loss of monomers (via their conversion to dimers, oligomers, proto-fibrils and fibrils), starting from a known concentration of the amyloid nomer. Importantly, by quantifying the intensity of the bands on the gel, it permits the user to build a kinetic profile of the consumption of monomers, formation of dimers, oligomers and finally the transformation of the amyloid protein into mature fibrils. From a biomedical perspective, the use of PAGE to establish a quantitative and dose-dependent profile of small-molecule efficiency in dissolving fibrils and oligomeric aggregates to their monomeric counterparts is highly desired.

In conclusion, we demonstrate that a readily existing method and easily accessible appartatus can be used to obtain rich biophysical (kinetic) data about amyloid forming trajectories and the interplay between intermediates therein. Equally importantly, it can be used to screen small-molecules and also determine, via size analysis, where along the trajectory that the small-molecule intervenes. It provides undergdatuates, graduate students and advanced biomedical researchers in an insittituion with a powerful, affordable, facile method, which is already widely availble, to study an important neurodegeneration-associated process.
